# Observations and Cases Illustrating the Efficacy of Compressing the Carotid Arteries in Sanguineous Turgescence of the Cerebral Vessels

**Published:** 1819-04-01

**Authors:** M. Blaud


### *Observations and Cases illustrating the Efficacy of compressing the Carotid Arteries in Sanguineous Turgescence of the Cerebral Vessels.* By M. Blaud, M.D.

I have had frequent opportunities of observing a cerebral affection in children, which sometimes comes on very suddenly, and without any premonitory symptoms—sometimes exhibiting a few precursory signs. In the first case, the child falls all at once into the most profound sleep, from which nothing can rouse him. In the second species, he feels a tingling sensation or numbness of one of the arms, and sometimes in the corresponding side of the face. He is generally frightened by these symptoms, and calls for assistance; but soon becomes embarrassed in his speech, and shortly afterwards falls into a state of insensibility.

Sometimes the disease is announced by delirium; great agitation; subsultus tendinum; convulsive movements; tetanic stiffness of the voluntary muscles; impeded respiration; partial paralysis, &c.

When the affection is completely developed, there is total immobility and insensibility; the countenance is flushed—sometimes, however, not very different from health, but it is at others convulsed; the eyes protruded, sparkling, and their vessels injected—pupils sometimes dilated, sometimes not; respiration at one time natural, at another stertorous; the pulse frequent, strong, full, but gradually becoming feeble; the breathing becomes more and more difficult; the face livid or pale, and death ends the scene.

The duration of the disease is only a few hours from the first moment of seizure. Sometimes death is almost instantaneous, as from syncope; and without any premonition from the pulse, countenance, or breathing.

The complaint is almost always fatal, and dissection shews a morbid condition of the cerebral vessels, more or

---

bies Contagiosa, accompanying the above valuable paper, as they only depict the usual features of that terrible disease. The paper itself will be read with no common interest; and the prophylactic measure, which rests on such a firm basis of experience—an experience which can fall to the lot of few individuals, must be duly appreciated by an enlightened profession.

Editor.

less intense. But a few cases will complete the description, and evince the nature of this dangerous malady.

Case 1. In the month of September, 1805, I was summoned to the assistance of a girl of twelve years of age, who, previously in health, and after having passed a good night, was suddenly seized, and that from no evident cause, with an alarming comatose affection. She had got up at seven o'clock in the morning, in perfect health, and about half an hour afterwards she felt a weight in her head, accompanied with such a degree of somnolency as forced her to lie down again, where she immediately fell into a profound sleep, from which nothing could rouse her. There was a total loss of power over the voluntary muscles, as in complete paralysis; insensibility; pulse somewhat quickened; respiration easy; pupils dilated, and insensible to the light; expression and colour of the face natural; heat of the surface moderate.

Aspersions of cold water on the face and trunk had no effect; sinapisms to the feet, and leeches to the neck had no sooner begun to operate than the countenance changed and turned pale all at once; the lips became livid; she foamed at the mouth; the pulse sunk, and was imperceptible. She expired at nine o'clock the same morning.

*Dissection, twenty-seven Hours after Death.*—The cerebral veins and sinuses gorged with blood; surface of the brain red, and its vessels injected; numerous red points throughout the substance of this organ. In other respects the organization sound.

Case 2. On the 6th of December, 1814, the parents of a child, five years old, and who had hitherto enjoyed good health, perceived its legs œdematous, and its abdomen larger than natural. I was desired to see the child, and easily recognized an ascites, without any perceptible organic lesion. The child was put upon a course of squills and calomel. The urine began to flow more copiously, and the dropsy had almost disappeared, when, in the night of the 13th, the child, after drinking, of its own accord, a glass of cold water, was suddenly seized with delirium and great difficulty of breathing, (the respirations being sixty in the minute) with small, weak, and frequent pulse; pale face; livid lips; great alteration of features; subsultus tendinum; wild ravings; constant efforts to get out of bed; pupils dilated, and little sensible to light,

*Treatment.* Blisters to the nape of the neck; antispasmodics; sinapisms to the feet, legs, and thighs.

13th. In the same state.—At 5 in the evening, general convulsions; tetanic stiffness of the whole body; trismus; and, all at once, loss of sense; coma; quicker respiration [80 in the minute]—At 7 o'clock, Rattling in the throat; body covered with livid spots; trismus and tetanus continue; pulse imperceptible; death at eight o'clock.

*Dissection, thirty Hours after Death.*—Cerebral veins gorged with blood; surface and substance of the brain more red than natural; about an ounce of turbid serum at the base of the skull; nearly the same quantity in the ventricles; lungs sound, crepitous, and of a pale yellow colour; right ventricle of the heart distended by a clot of blood; left ventricle empty. About eight ounces of serum in the cavity of the abdomen; all the other organs sound.

Case 3. On the 3d of February 1817, a child, seven years of age, in good health, and rather embon point, was suddenly seized in the afternoon, with convulsive motions of the right upper and lower extremities. In a few minutes afterwards, stammering came on, and ultimately loss of speech and sense; stertorous breathing, without any alteration in the countenance. The limbs continued to be agitated with convulsive motions. This state lasted an hour. The child came to itself again, but with loss of speech, and a heavy stupid look. In half an hour, the powers of speaking returned; the convulsive movements ceased, when complete paralysis supervened. In about two hours, the paralysis also disappeared spontaneously.

4th. Nasal hæmorrhage.—In the evening, head ache, which was removed by a short sleep. From this time till the 14th, the child complained every evening of a heavy pain in the fore part of the head, attended by drowsiness.

14th. At two o'clock in the afternoon, shiverings, headache, drowsiness, and a short sleep. At half past four o'clock, on awaking, the child was all at once seized with subsultus tendinum of the right arm, shortly afterwards followed by paralysis. In a few seconds the speech became confused, with loss of sense, locked jaws, stertorous breathing, convulsions, fixed eyes.

We were called in consultation at eleven o'clock at night, and found the child in the following state: Face flushed; expression natural; eyes moving about; pupils very dilated, and insensible to the light; convulsive movements of the muscles of the right side; the body otherwise immoveable; insensibility; coma; stertorous respiration; foaming at the mouth; pulse small, irregular, and innumerable.

The disease had been mistaken for a *nervous* affection, and antispasmodics had been exhibited in abundance, but without producing any good effect. Leeches, blisters, &c. were now prescribed, but it was too late; the child expired at four in the morning.

*Dissection.* — Cerebral veins and sinuses gorged with blood; the whole surface of the brain red, and highly injected. The sliced surfaces of the brain exhibited numerous red points; the same in the cerebellum. Other organs sound, except that the cells of the lungs were filled with viscid serum.

The foregoing Cases will convince us that the disease in question consisted in a sanguineous congestion of the cerebral vessels, determined, no doubt, by an irritation inducing a super-excitement of the vital powers of the brain. If this view be correct, it follows that the most prompt and powerful mean of arresting the progress of the malady, would be such as quickly extinguishes this morbid sensibility in the brain, by suspending the current of arterial blood to that organ; on which blood its excitement depends.

The following Cases will elucidate this reasoning.

Case 4. On the 26th of June, 1818, at six o'clock in the morning, Margaret Artand, five years and a half old, who had been in previous good health, complained of the left arm and left side of the face, without being able to describe its feelings; the fingers of that hand were observed to be powerless. After continuing six hours in this state, there was sudden loss of sense and speech; face drawn to one side, red, swelled, of a violet colour; left arm convulsed. She was put to bed, and various means were used to rouse her from this state, but all to no purpose. At eight o'clock the convulsive movements ceased, the coma continuing, with complete insensibility and immobility. I was called to her at half past nine, and found her with the following symptoms: Face swelled, and red; eyes half open, and fixed, prominent and injected; complete coma and insensibility, even to the pricks of needles; the respirations forty in the minute; pulse 130, strong, full; temporal and carotid arteries throbbing violently; skin intensely hot with some moisture.

The Case was urgent; blood-letting strongly indicated, but we had no instruments for the purpose. We immediately compressed with our fingers and thumb the carotid arteries. In seventeen seconds the child began to move, all at once; and raising its left hand, thrust off our fingers from its neck with great violence, and thus removed the compression from the carotids. The child quickly fell into the same state of coma as before. We renewed the compression, and in thirteen seconds the child suddenly started on its breech, to the great astonishment of the bye-standers; opened its eyes; called out for its mother; and defended itself so strongly against our efforts, that the compression was once more removed. For a few minutes the child sat up, viewing us with terror, and calling loudly for its mother; when gradually the eyes became dull, the eye lids dropped, and the little patient fell back motionless in its bed.

Our former success urged us to a repetition of the experiment. At the ninth second of the third compression, the child came suddenly to her senses, as before, raised herself on her breech, and called out for her mother. Notwithstanding her struggles, we succeeded this time in keeping up the compression for 25 seconds. The coma or insensibility did not, this time, return. The child became perfectly sensible; demanded drink, and knew all the bye-standers. She complained only of pain in the forehead. We repeated the compression for a few seconds at a time, every half hour, till two o'clock in the morning. At three o'clock the patient fell into an easy tranquil sleep, from which she was readily roused, complaining only of head-ache, which was completely removed in the course of the next day by glysters, and the warm mustard bath to the feet and legs.

Case 5. Francis Niquet, 19 years of age, a farmer, complained on the 10th of August, 1818, of acute head-ache, especially in the frontal region, which continued till four o'clock in the afternoon of the next day, when he lay down in his own hay-loft. At eight o'clock his mother found him insensible and motionless; his face flushed, of a violet colour, swelled; the eyes half open, blood-shot, and prominent; the respiration hurried. M. Linné, a surgeon, was called, and bled the patient copiously from the arm, but without any good effect. Recollecting the success which attended our compression of the carotid arteries, on a former occasion, he determined on putting it to the trial in this case. He immediately compressed firmly both the carotids. In 30 seconds the face resumed its natural colour; the patient started on his breech, recovered his speech, and angrily demanded what the Surgeon was about, forcing the fingers of the latter from their position on his neck. But by little and little the comatose affection returned, and the patient fell again into the same state from which he had just emerged. The carotids were again compressed, and in a few seconds the power of motion returned, with sense and speech. M. Linné was again forced to abandon the compression, in consequence of the patient's violence, and again the fit returned, but not in near so violent a degree. Finally, after the fourth compression there was no further attack. The patient continued perfectly well, excepting a slight head-ache, which was removed by a few leeches to the temples.

---

